# Do State Comprehensive Planning Statutes Address Physical Activity?: Implications for Rural Communities

**DOI:** 10.3390/ijerph182212190

**Published:** 2021-11-20

**Authors:** Lisa M. Charron, Chloe Milstein, Samantha I. Moyers, Christiaan G. Abildso, Jamie F. Chriqui

**Affiliations:** 1 Nelson Institute for Environmental Studies, University of Wisconsin-Madison, Madison, WI 53706, USA; 2 School of Public Health, University of Illinois at Chicago, Chicago, IL 60612, USA; calong@uic.edu (C.M.); jchriqui@uic.edu (J.F.C.); 3 Department of Social and Behavioral Health Sciences, School of Public Health, West Virginia University, Morgantown, WV 26506, USA; smoyers2@hsc.wvu.edu (S.I.M.); cgabildso@hsc.wvu.edu (C.G.A.); 4 Institute for Health Research and Policy, University of Illinois at Chicago, Chicago, IL 60612, USA

**Keywords:** physical activity, rural, policy, comprehensive plan, built environment, urban planning, state statute, legal epidemiology

## Abstract

Less than one-quarter of U.S. adults meet physical activity (PA) recommendations, with rural residents less likely to be active than urban residents. The built environment has been identified as a potential facilitator of PA and local comprehensive plans are a foundational tool for guiding the development of the built environment. The purpose of this study was therefore to understand the current landscape of comprehensive planning state statutes related to PA and rural communities. We used primary legal research methods to identify, compile, and evaluate all 50 state comprehensive planning statutes for items related to PA and conditional mandates based on population size of local jurisdictions. The presence of population-conditional planning mandates and the inclusion of PA-related items was analyzed by state-level rurality using Fisher’s exact tests. Our analyses demonstrated that (1) broader PA-related items were addressed in state statutes more often than more specific PA-related items; (2) when PA-related items were addressed, they were most likely to be mandated, subsumed elements; (3) several PA-related items were less likely to be addressed in the most rural states and/or conditionally mandated for jurisdictions meeting minimum population requirements; and (4) only two states addressed PA directly and explicitly in their comprehensive planning statutes.

## 1. Introduction

Physical activity (PA) provides countless mental health [1] and physical health [2,3] benefits, but only 22.8% of adults in the United States (U.S.) meet the U.S. Department of Health and Human Service’s (DHHS) PA guidelines for achieving those health benefits [4]. Although this figure has improved over recent years, rural residents lag behind urban residents: 19.6% of rural residents and 25.3% of urban residents meet DHHS PA guidelines [5]. While a wide array of PA barriers and facilitators have been reported in the literature [6,7,8], a systemically equitable [9] and potent facilitator [10] is a built environment conducive to PA. 

Socioecological theory outlines the crucial role of the policy and built environment in creating active communities [11,12,13]. Creating PA-friendly environments has become a priority for reaching global public health, economic development, and sustainability goals [12,14,15,16]. Researchers have identified many specific built environment features and characteristics associated with increased PA [17,18,19], including bicycling infrastructure [20], mixed-use land development [19,21], increased residential density [19], and access to parks and recreation [19].

However, rural built environments may be less likely to promote PA than urban ones. Rural environments present unique barriers to PA, including lower residential and job density, fewer facilities for recreational PA and lack of public transportation to and from such facilities, fewer destinations in town centers, lack of pedestrian infrastructure, high-speed and heavy commercial traffic, and fear of wild and domestic animals [22,23,24,25,26]. Despite the unique environmental barriers to PA that rural residents face and the urban–rural disparity in PA, research on planning for active living has largely focused on urban environments [27].

Local governments—whether in urban, suburban, or rural communities—are uniquely situated to facilitate the development of healthy built environments through local planning and built environment policies. In collaboration with local residents, policymakers, and other stakeholders, local planners (e.g., planning consultants, staff planners, regional planners) define a vision for the development of a community’s built environment and identify goals, policies, and actions that the community can take to make that vision a reality [28,29]. Such policies and actions might include the creation of community design guidelines, tax incentive programs for certain types of development, and capital investment plans, among others. This vision and the ensuing goals, policies, and actions are often encoded in a comprehensive plan [29,30,31].

Comprehensive plans cover a wide range of topics, including transportation, economic development, and housing. They are often considered the foundational document of local planning practice, making them important policy levers for addressing cross-departmental, intersectoral, and systemic issues like health equity and PA promotion [28,32,33]. In many cases, comprehensive plans directly impact built environment policies like zoning codes through consistency requirements [34,35].

The literature evaluating PA-promoting components of comprehensive plans is still emerging. In 2010, an American Planning Association (APA) survey of 890 local planners and officials found that 57.1% of adopted comprehensive plans addressed active living in some way [36]. A 2014 survey of local officials in the U.S. found that approximately three-fourths (78%) of comprehensive plans included at least one of three active living goals or objectives: implementation of a Complete Streets policy, development of street connectivity, or encouragement of mixed-use development [37]. Several studies have found that active living policies and programs are enacted more consistently when they are included in comprehensive plans [38,39], and others have shown that the incorporation of active living components in comprehensive plans and zoning ordinances is associated with higher PA levels, reduced PA disparities, and even lower cancer incidence [9,40,41,42,43,44,45].

Comprehensive plans may be particularly powerful tools for rural communities to address lack of PA through built environment interventions; however, comprehensive plans are less prevalent and less likely to include PA-promoting goals and policies in rural communities [37]. One reason for this may be variation in how state comprehensive planning statutes address rural communities and PA. While the specific content (and presence) of a comprehensive plan is largely driven by local leadership and public input, state statutes enabling comprehensive planning also play a role. The Standard City Planning Enabling Act (SCPEA) of 1927 attempted to standardize state planning statutes; however, it was not uniformly adopted by the states [46]. Other model planning laws and regulations, or portions thereof, have also been adopted by various states over the years [46,47,48]. This has resulted in wide variability in comprehensive planning enabling acts among states, both in terms of required planning processes and required plan content. Strong state comprehensive planning mandates are associated with the presence of a locally adopted comprehensive plan and higher-quality comprehensive plans [49,50].

To our knowledge, an up-to-date evaluation of comprehensive planning state statutes focused on components that are likely to promote PA does not exist. The APA has surveyed state comprehensive planning enabling statutes with a focus on requirements for hazard mitigation [34] and housing [51] elements. In addition, the Growing Smart Legislative Guidebook, published by the APA in 2002, included an evaluation of state comprehensive planning statutes. This evaluation addressed how much statutes differ from the SCPEA; whether the statutes mandate, conditionally mandate, or encourage comprehensive planning; the extent to which statutes address 20 types of broad plan elements, including land use, recreation, transportation, and historic preservation; and the strength of the state’s role in supporting local planning [47]. This evaluation also noted to which types of jurisdictions (e.g., towns, villages, cities, counties) comprehensive planning mandates are applied [47]. However, past evaluations have not analyzed state statutes with a focus on implications for rural communities.

The purpose of this study was therefore to understand the current landscape of comprehensive planning state statutes related to PA and rural communities. This study seeks to characterize the extent to which state comprehensive planning statutes address PA-related elements and topics, if and how the laws apply differently to rural versus urban communities, and how these characteristics vary by state-level rurality. To our knowledge, this is the first comprehensive 50-state evaluation of the statutory requirements for local plans to include information on transportation, land use, parks and recreation, PA, and equity, and to focus on implications for comprehensive planning in rural communities.

## 2. Methods

### 2.1. State Statute Identification and Coding Protocol

We used primary legal research methods [52,53] to identify and compile codified state statutory laws for each of the 50 states using state law databases available via the commercial legal research service LexisNexis [54]. Following well-established policy surveillance methods [55], we systematically searched each state’s statutes using Boolean keywords as well as line-by-line reviews of the indices and tables of contents to locate relevant components of the codified statutes that were on-the-books as of January–February 2021.

A detailed coding protocol was established to guide the review and coding of each state’s statutes and to ensure consistency in the process. While coding, we tracked ambiguities in the language of the state laws and met regularly to discuss such instances. We reached consensus in coding decisions based on the letter of the law, combined with the team’s expertise in planning, land use, and PA-related issues. All such decisions were documented, and state laws were re-reviewed in instances where a decision led to a potential for inconsistency across states. Where possible, we also verified the contents of state laws against a publicly available secondary source from the APA [34], which contained historical information on relevant state laws, and previously compiled but unpublished data from a member of the study team (J.F.C.).

We excluded codified state laws from the analysis that addressed non-mandatory regional and/or joint planning and home rule charters. Due to resource limitations, we excluded codified state regulations—even if they were embedded by reference into a codified state statute—and non-codified state policies. One study author (C.M.), a trained legal researcher, reviewed all relevant state laws under the direction of another study author (J.F.C.) with extensive policy surveillance experience.

### 2.2. State Statute Variables

Several characteristics of the state statutes were evaluated with regard to the current study: (1) requirements or encouragement to include any of 19 items related to PA in comprehensive plans (described in more detail in the following sub-sections and listed in Appendix A); (2) whether these items were discussed as primary elements, subsumed elements, or topics; and (3) the nature of conditional mandates to develop comprehensive plans and to include items related to PA in the plan.

#### 2.2.1. Required or Encouraged Items Related to PA

The state laws were qualitatively reviewed for whether and under what circumstances a county or municipality is required to include in its plan information on the following 19 items: PA, equity, bicycle/pedestrian, bicycling, pedestrian, public transportation, land use, streets, transportation/circulation, design, infill/reuse, mixed use, smart growth, farmland preservation, historic preservation, parks/recreation, open space, trails, and natural resources. A list of PA-related comprehensive plan items was developed by consulting two validated comprehensive plan toolkits of evidence-informed active living strategies, “Healthy Living and Active Design: A Scorecard for Comprehensive Plans” [56] and “Healthy Rural Community Design: A Scorecard for Comprehensive Plans” [57]. This list was refined to the final list of 19 items through discussion with the research team. A detailed codebook for these items is included in Appendix A.

Each item that was included in a state law was also coded by the strength of the requirement—as mandated (strongest), conditionally mandated, or encouraged (weakest). Items required by law to be included in a plan were coded as mandated. Items required by law to be included in a plan if certain criteria were met (e.g., population minimum) were coded as conditionally mandated. Items not required by law to be included in a plan, but that were mentioned in the law or suggested to be included, were coded as encouraged.

For each of the 19 PA-related items, a four-level variable was created with values “Mandated”, “Conditionally mandated”, “Encouraged”, and “Not Addressed”. A binary summary variable was also created, taking values “Yes” (the item was addressed in the state statute as mandated, conditionally mandated, or encouraged) and “No” (the item was not addressed in the state statute). Each of the 19 items could be included in multiple sections of a state statute and thus coded multiple times. We retained only the instance with the strongest requirement. For example, a state statute could have mandated the item “bicycling” in one section and also encouraged it elsewhere; it would have been coded only once for that state, as “mandated”.

#### 2.2.2. Primary Elements, Subsumed Elements, and Topics

In addition to whether PA-related items were mandated, conditionally mandated, or encouraged, we wanted to know whether these items were discussed as primary elements, subsumed elements, or as topics. For each of the 19 PA-related items that we initially coded as included in the statute, we further coded the item as a “primary element” if it was treated by state law as a distinct section of a plan (either through use of language such as element, plan, chapter, component, or section; or through placing the item in a list of other items referred to as elements, plans, etc.). We coded the item as a “subsumed element” if it was treated by state law as information to include within or as part of a primary element. For instance, for a law providing that a plan shall include “a land use element designating the proposed general distribution and general location and extent of the uses of land…for…recreation, open spaces…”, land use was coded as a primary element, and parks/recreation and open space were each coded as subsumed elements. We coded the item as a “topic” if a state law discussed its inclusion in a plan, but not as a primary or subsumed element. For instance, for a law providing that the plan “may provide for…energy conservation, transportation… and recreational…opportunities”, conservation/natural resources, transportation, and parks/recreation were coded as topics. For each of the 19 PA-related items, three binary variables were created (which took the values “Yes” and “No”), representing the categories: primary element, subsumed element, and topic.

#### 2.2.3. Conditional Mandates

The state laws were qualitatively reviewed for whether and under what circumstances a county or municipality is required to develop and adopt a comprehensive plan. Laws that required a county or municipality to plan or provide for planning if certain criteria were met (e.g., population minimum) were coded as conditionally mandated. The nature of each conditional mandate was then evaluated and thematically coded into broad categories by the first author (L.M.C.). Binary variables were created for each of several conditions put on mandates to develop a comprehensive plan (e.g., population minimum, presence of a plan commission) that took the values “Yes” and “No”. We primarily present results for population-based conditional mandates because this is the closest proxy for conditional mandates based on rurality of local jurisdictions.

Binary variables were also created for each PA-related item representing the presence (“Yes”) or absence (“No”) of a population-conditional mandate to include that item.

### 2.3. State-Level Rurality

State-level rurality was defined as the percent of the state population living outside of a 2010 U.S. Census Bureau defined Urban Area [58], from which a categorical variable was created using tertiles. These categories are labelled “Least Rural” (*n* = 16, ≤16.70% rural population), “Mixed Rural/Urban” (*n* = 17, 16.71–33.67%), and “Most Rural” (*n* = 17, ≥33.68%).

### 2.4. Data Analysis

Descriptive statistics were obtained for the characteristics of interest based on the review and coding of the state statutes. We analyzed the number and percentage of states that mandated, conditionally mandated, and encouraged the inclusion of each of the 19 PA-related items; the number and percentage of states that addressed each of the 19 PA-related items as primary elements, subsumed elements, and topics; the number and percentage of states that had a mandate for comprehensive planning conditioned on the population of the local jurisdiction; and the number and percentage of states that had population-conditional mandates for any of the PA-related items. Furthermore, among states that addressed each of the 19 PA-related items, we calculated the percentage that had population-conditional mandates.

The presence of population-conditional planning mandates and the inclusion of PA-related items was further analyzed by state-level rurality. Fisher’s exact tests were used to test associations between the binary state statute variables (presence or absence of a certain characteristic) and state-level rurality. The Fisher’s exact tests in this study test the null hypothesis that the percentage of states that have the characteristic in question does not differ between the three levels of rurality. Although these data represent a census rather than a sample, Fisher’s exact tests are appropriate because of the small number of observations and small expected values in the contingency tables [59]. All data analysis was conducted using Stata 16.1 (StataCorp, College Station, TX, USA) [60].

## 3. Results

All 50 states had laws regarding comprehensive planning for counties, municipalities, or both. Two states, Georgia and Oregon, did not discuss any required or encouraged items in their comprehensive planning state statutes; however, these states are retained in the denominator of the data because they did have comprehensive planning statutes on the books.

### 3.1. Requirements and Encouragement for Items Related to PA

The prevalence of PA-related items in the state statutes varied widely, as shown in Table 1.

Five items were addressed in at least 70% of state statutes: parks/recreation was discussed in 90% of states, land use in 88%, transportation/circulation in 80%, streets in 74%, and natural resources in 74%. On the other hand, eight items were addressed in less than 25% of the state statutes: PA (4% of states), smart growth (8%), bicycle/pedestrian (14%), infill/reuse (16%), mixed use (16%), pedestrian (18%), trails (20%), and bicycling (22%). With a few exceptions, when items related to PA were addressed in state statutes, they were most likely to be mandated rather than conditionally mandated or encouraged. The items most frequently mandated were land use (64% of states), transportation/circulation (50%), parks/recreation (46%), and streets (44%). As compared to mandated and encouraged items, relatively few states conditionally mandated items, with the notable exception of the transportation/circulation item (24% of states conditionally mandated).

Table 2 presents the 19 items of interest displayed by element/topic types rather than level of mandate (and, unlike the categories in Table 1, the categories in Table 2 are not mutually exclusive).

Most of the PA-related items were more likely to be addressed in state statutes as a subsumed element rather than a primary element or a topic. The notable exceptions were transportation/circulation, land use, and natural resources, which were more likely to be addressed as primary elements; and equity, which was more likely to be addressed as a topic. Many PA-related items addressed as subsumed elements were subsumed under several different primary elements. Often, PA-related items were addressed as subsumed under primary elements that were outside the scope of this analysis (i.e., primary elements not related to PA).

### 3.2. Differing Comprehensive Planning Mandates Based on Local-Level Rurality

**Figure 1:**
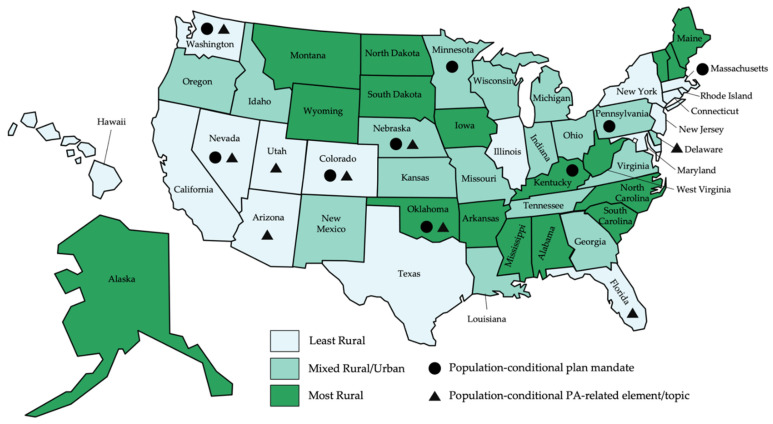
Map of state-level rurality with presence of population-conditional rules for comprehensive planning and addressing PA-related plan elements or topics.

The closest indicator of differing comprehensive planning requirements for rural versus urban communities we observed in the state statutes was conditional mandates based on population of the county or municipality. Out of the 50 states, only five—Colorado, Nevada, Washington, Oklahoma, and Nebraska—had population-conditional mandates for both comprehensive planning and for PA-related elements/topics. Four states—Massachusetts, Kentucky, Pennsylvania, and Minnesota—had population-based conditional mandates only for comprehensive planning generally, while four other states—Delaware, Florida, Arizona, and Utah—had population-based conditional mandates for PA-related plan elements/topics only. The language for each of these state’s population-conditional laws can be found in Appendix B. Figure 1 visualizes these results and shows that the states with population-conditional mandates are diverse with regard to geographic location.

There was no discernible pattern to population-conditional mandates for comprehensive planning based on state-level rurality, though there was for population-conditional mandates for PA-related items. Of the nine states with population-conditional mandates for incorporating PA-related items, six (66.7%) were in the least rural states. The Fisher’s exact test for an association between state-level rurality and population-conditional mandates on PA-related items yielded a *p*-value of 0.072.

Some PA-related items were more likely to have mandates based on population size than others. Table 3 shows the prevalence of states with population-conditional mandates regarding each item (col 2) as compared to the number of states that addressed the item at all in their planning statutes (col 3). It also includes the percentage of states with population-conditional mandates for each item over the total number of states that address that item (col 4).

In absolute terms, the items that were discussed in the greatest number of state statutes were also the ones that had population-conditional mandates in the greatest number of states. These include parks/recreation (seven states with conditional mandates), open space (six), land use (six), transportation (five), and streets (five). However, for five items, at least one in five states that addressed the item put population-based conditions on requiring that item in comprehensive plans: bicycle/pedestrian (28.6%), mixed use (25.0%), smart growth (25.0%), open space (20.0%), and trails (20.0%).

### 3.3. Differences in PA-Related Elements and Topics Based on State-Level Rurality

Table 4 shows the prevalence and percent of states addressing PA-related comprehensive plan items by state-level rurality. The table also shows the result of the Fisher’s exact test for any relationship of each item with state-level rurality.

For most items, there is no statistical relationship. That is, the rurality of a state’s population is not associated with the inclusion of many PA-related items in comprehensive planning statutes. There are, however, a few exceptions. For transportation/circulation (*p* = 0.038), public transportation (*p* = 0.074), mixed use (*p* = 0.001), infill/reuse (*p* = 0.023), smart growth (*p* = 0.008), and PA (*p* = 0.098) there was an association between the item and state-level rurality. Most of these items were addressed most frequently in the least rural states and equally infrequently in the mixed rural/urban or most rural states whereas public transportation was addressed less frequently with increasing rurality.

## 4. Discussion

PA provides many health benefits and local governments are well-situated to facilitate such activity through thoughtful comprehensive planning intended to create environments conducive to PA. The process and content of comprehensive planning is guided by state statutes that vary across the 50 states in the U.S. This study sought to evaluate state-level policies guiding the process of local comprehensive planning and the PA-related content of those plans, with particular attention paid to implications for rural jurisdictions.

### 4.1. Findings

This is the first 50-state review of the statutory requirements for local comprehensive plans to include information on transportation, land use, parks and recreation, PA, and equity, and the first review of state statutes to focus on differing comprehensive plan requirements by rurality. Our analyses demonstrated (1) broader PA-related items were addressed in state statutes more often than more specific items directly related to PA; (2) when PA-related items were addressed, they were most likely to be mandated, subsumed elements; (3) several PA-related items were less likely to be addressed in the most rural states and/or only conditionally mandated for jurisdictions meeting minimum population requirements; and (4) only two states addressed PA directly and explicitly in their comprehensive planning statutes.

#### 4.1.1. PA-Related Items in State Statutes

When states did address items related to PA in their comprehensive planning statutes, they were more likely to address broad topics than specific strategies to promote PA. Of the 19 items analyzed, the most commonly required or encouraged items relevant to PA were parks and recreation (90% of states), land use (88%), transportation (80%), streets (74%), and natural resources (74%). State statutes can facilitate long-term built environment change in communities by mandating or encouraging the inclusion of PA-related items in comprehensive plans. Built environment approaches that combine transportation and land use changes are recommended by the Community Preventive Services Task Force, and the Task Force encourages planners to consider such strategies [19]. However, apart from parks and recreation, the PA-related items most likely to be addressed in statutes are so broad that they could be included in a plan without encouraging PA at all. For example, a community could include a transportation or streets chapter in their comprehensive plan that focuses completely on automobiles to the exclusion of bicyclist and pedestrian transportation considerations. Land use could be discussed in such a way that promotes segregated land uses and suburban sprawl rather than mixed-use and compact development. Many of the infrastructure and planning strategies that have been more specifically and directly linked to promoting PA [18,19,22,61,62,63,64,65] were required or encouraged in less than one quarter of state statutes: smart growth (8%), bicycle/pedestrian (14%), infill/reuse (16%), mixed use (16%), pedestrian (18%), trails (20%), and bicycling (22%).

When they were addressed at all, most of the PA-related items were more often addressed as subsumed elements rather than as primary elements or topics. Most of the PA-related items we analyzed may simply be too narrow to merit their own chapter or section of a comprehensive plan; indeed, the items that were more likely to be included as primary elements are some of the broadest of the items (i.e., transportation/circulation, land use, and natural resources). It is encouraging that when the PA-related items were addressed in state statutes, they were most often mandated rather than conditionally mandated or encouraged. This indicates that, while many of the PA-related items may not be deemed broad, complex, or important enough to merit their own chapter or section of the comprehensive plan, they still merited a mandatory sub-section of a chapter.

The notable exceptions to this typology were public transportation, farmland preservation, design, mixed use, and trails, which were all more likely to be encouraged than mandated or conditionally mandated; and equity, which was more often mandated, but as a topic rather than a subsumed element. These are important areas for PA-promotion and states may therefore consider being more prescriptive on these elements in state statutes.

#### 4.1.2. Comprehensive Planning and PA-Related Items by State- and Local-Level Rurality

Six items were less likely to be addressed by statutes in states with higher rural populations: transportation, public transportation, mixed use, infill/reuse, smart growth, and PA. In addition, at least one in five states that addressed several of the PA-related items had population-minimum requirements for including that item in local comprehensive plans: bicycle/pedestrian (28.6% of state statutes that addressed the item had a population-minimum mandate), mixed use (25.0%), smart growth (25.0%), open space (20.0%), and trails (20.0%). These items, therefore, may be less likely to be addressed in rural, local comprehensive plans across the U.S.

There may be the notion among policymakers and planners that these PA-related items are not relevant to rural places. However, public transportation is particularly important in rural communities, which have an increasingly large share of older (65+) residents who may have trouble accessing community services and resources [66,67]. While it may seem that rural residents have the private recreational resources (i.e., large lots) necessary to be physically active, studies have found that lack of access to public spaces for recreation and social isolation are barriers to PA in rural communities [22,27]. Therefore, trails, open spaces, and other public places for PA may be especially important facilitators in rural communities. Trails, moreover, have been identified as one of the most effective strategies for rural communities to promote active transportation and recreation, while also building recreational tourism [22,27,68,69]. Lastly, when implemented in a context-sensitive way, mixed use, infill/reuse, smart growth, and open space planning can help rural communities preserve rural land, control infrastructure costs, and maintain a small-town character and sense of community, while also promoting PA [57,68]. Along with the built environment strategies discussed above, PA itself is important for rural communities to explicitly address comprehensive plans because it is less prevalent in rural adults than urban adults [5,70].

Thirteen states (26%) had conditional mandates based on population for local-level comprehensive planning (four states), for the incorporation of any of the PA-related items in local comprehensive plans (four states), or both (five states). Population-based conditional mandates for PA-related items (but not for comprehensive planning in general) were more likely in the *least rural* states and they varied greatly in their complexity. For example, Delaware’s population-conditional mandate for PA-related items is for municipalities with 2000 or more population. On the other hand, Arizona had different population thresholds for different items, ranging from 2500 to 200,000+ population minimums. Several states combined their population-based mandate with another condition. Arizona, Colorado, and Washington had multi-part conditional mandates based on a combination of minimum population size and minimum population growth rates. Kentucky, Massachusetts, Minnesota, Nevada, Oklahoma, and Washington combined population-based conditions and conditions based on the presence of a planning commission or agency. Often, comprehensive planning and/or the inclusion of PA-related items was encouraged for jurisdictions in which it was not mandated. Other states had standalone conditional mandates based on the presence of a planning body; the desire to zone; minimum population growth rates; if the jurisdiction’s comprehensive planning process was funded by a state grant; and vague conditions like “where pertinent”, “as appropriate to the municipality”. These conditions may be less likely to apply to rural communities as well as the population-minimum conditions (e.g., if the jurisdiction does not have the capacity to staff a plan commission or if they choose to not zone because of lack of development pressure).

Rural communities may have less capacity and funding to prepare a comprehensive plan [57,71] and states with higher rural populations may face political opposition to strong comprehensive planning laws, particularly ones that advocate for policies like smart growth [72]. Conditional mandates based on population and the fact that the most rural states were less likely to address several PA-related items might stem from these challenges. However, comprehensive plans can be powerful tools for communities to determine the direction of future development and to address, in a proactive and context-sensitive way, their most pressing social, economic, and health challenges. Allowing rural communities to opt out of comprehensive planning or including PA-related items in their comprehensive plan does not set them up to face these challenges and may even contribute to the rural–urban PA disparity [5].

PA and built environment strategies that promote PA are just as important in rural communities as they are in urban ones. Addressing PA-related items in state comprehensive planning statutes could create important local-level policy and built environment changes, and these changes should not be limited to urban areas. However, states should also be aware that planning for PA is a context-sensitive practice; built environment policies to promote PA will look different in rural versus urban communities [27]. Local governments are well-situated to understand and address the unique needs of their individual communities, but they may need a push from state law to consider certain topics in their comprehensive plans. Therefore, comprehensive plan enabling state statutes should strike a balance between (a) mandating inflexible PA-promoting planning policies that are neither appropriate, effective, nor feasible in rural communities, and (b) offering so much flexibility (or mandates conditioned on population) that rural communities omit policies that can promote PA. Creating technical assistance programs and grant funding for comprehensive planning processes may also help rural communities meet such mandates.

#### 4.1.3. PA Language in State Statutes

PA itself was only discussed in two states. We quote these state statutes below in order to provide instructive examples of policy language. California’s state statutes conditionally mandated a subsumed element about PA for both county and municipal comprehensive plans, under a mandated primary “environmental justice element”. They stipulated: 

“(h)(1) An environmental justice element, or related goals, policies, and objectives integrated in other elements, that identifies disadvantaged communities within the area covered by the general plan of the city, county, or city and county, if the city, county, or city and county has a disadvantaged community. The environmental justice element, or related environmental justice goals, policies, and objectives integrated in other elements, shall do all of the following:

(A) Identify objectives and policies to reduce the unique or compounded health risks in disadvantaged communities by means that include, but are not limited to, the reduction of pollution exposure, including the improvement of air quality, and the promotion of public facilities, food access, safe and sanitary homes, and physical activity” [73].

Washington encouraged a subsumed element related to PA in both county and municipal comprehensive plans, under the conditionally mandated primary land use element. Its law stipulates: “Wherever possible, the land use element should consider utilizing urban planning approaches that promote physical activity” [74]. 

### 4.2. Strengths, Limitations, and Future Research

This study has several strengths. State statutes were identified through primary legal research methods, objectively coded by a legal researcher, and reviewed by the research team. Therefore, this study is not impacted by self-report or coder bias regarding the content of state statutes. In addition, we report information on a broad variety of PA-related items rather than a few items in one domain of PA (e.g., active transportation or parks). This allows us to take a broader view of PA and built environment aspects. Lastly, and perhaps most importantly, this study is novel. To our knowledge, this is the first 50-state review of the statutory requirements for local comprehensive plans to include information on transportation, land use, parks and recreation, PA, and equity, and the first review of state statutes to focus on differing comprehensive plan requirements by rurality.

Despite these strengths, there are several limitations to the present study. This is a cross-sectional study; therefore, relationships between state-level rurality and state statutes should be interpreted with caution. Conducting Boolean keyword searches of state statutes (combined with reviewing statutory indices and tables of contents, and continually reviewing coding protocol) is the best-practice method in policy surveillance that nevertheless has limitations [55,75]. There is the potential that we overlooked certain “wordings” that correspond to important PA-related content of the state statutes.

We only evaluated state comprehensive planning statutes and not regulations or government programs. Therefore, there may be other state-level laws (i.e., rules, regulations, and non-codified policies) regarding comprehensive planning that were not accounted for in this study. In addition, when evaluating comprehensive planning mandates and PA-related items, we did not take into account the diversity of consistency requirements, enforcement procedures, and encouragement mechanisms among states. These other state-level policies could impact the extent to which comprehensive planning mandates are taken up by local governments and therefore, should be the focus of future research. In particular, a combination of quantitative and qualitative research on differential enforcement and encouragement mechanisms for urban versus rural communities could shed light on how state-level comprehensive planning statutes translate to local-level planning efforts (and subsequent built environment changes) in different contexts.

Moreover, while there is evidence that strong comprehensive planning mandates do result in more and stronger comprehensive plans [50,76], state-level policies are only one factor influencing the presence and quality of local comprehensive plans. State and regional planning infrastructure, culture, and history, as well as local economic conditions, level of development pressure, capacity for planning, politics, and community support can all impact whether a comprehensive plan will be drafted and what it will contain. In addition, the extent to which comprehensive plans are implemented once adopted remains a question in the literature [30]. Therefore, more proximal research should be conducted to investigate when, how, and why rural jurisdictions incorporate PA-related items into their comprehensive plans, what role state comprehensive planning statutes play in the decision to include PA-related items, and what the long-term outcomes are of such incorporation (i.e., changes to the built environment, health behaviors, health outcomes). Lastly, longitudinal research could be targeted to the two states (California and Washington) that included PA in their comprehensive planning statutes to understand if this has led to PA being incorporated in local plans.

Our systematically-developed database of state comprehensive planning statutes can serve in the design and analysis of future research projects. In particular, comparative work based in different characteristics of state statutes should be conducted. This could help ascertain the role of state planning policy in influencing local-level planning policy, built environments, health behaviors, and health outcomes. For example, we found that several items most closely associated with PA promotion (smart growth, bicycle/pedestrian, infill/reuse, mixed use, pedestrian, trails, and bicycling) were encouraged or required in less than one-quarter of state statutes. Researchers could compare local-level comprehensive plans from states that do and do not mandate these items; this analysis could further be stratified by rurality to understand how state-level planning policy is implemented across contexts. In addition, researchers could compare the presence and quality of local-level plans across varying local contexts in states that do and do not have conditional mandates for planning and/or PA-related items.

## 5. Conclusions

Comprehensive planning can be a way for all communities—whether small town or large metropolitan—to develop a PA-friendly built environment. As states update comprehensive planning statutes, lawmakers should carefully consider including requirements for elements and/or topics specifically and directly related to the promotion of PA, or even, following the example of California and Washington, addressing PA itself. These requirements should apply to rural communities as well as urban ones, but they should be written in such a way that they can be applied in a locally-driven, context-sensitive manner. Comprehensive planning and PA-promoting built environment strategies may help rural communities not only address low community rates of PA, but also face their many social, economic, and health challenges. States may therefore also want to consider—and include language in their statutes pertaining to—the myriad co-benefits that are likely to arise from planning policies that promote PA, including economic, sustainability, community cohesion, and other health benefits [16,77]. Finally, states should strongly consider implementing technical assistance and funding programs to help rural communities develop comprehensive plans.

## Figures and Tables

**Table 1:** Prevalence and percent of states with statutes addressing, mandating, conditionally mandating, and encouraging comprehensive plan items related to physical activity (PA).

PA-Related Item	Addressed		Mandated		Conditionally Mandated		Encouraged	
	*n*	%	*n*	%	*n*	%	*n*	%
*Transportation*								
Transportation/Circulation	40	80	25	50	12	24	3	6
Streets	37	74	22	44	3	6	12	24
Public transportation	23	46	7	14	6	12	10	20
Bicycling	11	22	6	12	4	8	1	2
Pedestrian	9	18	6	12	2	4	1	2
Bicycle/Pedestrian	7	14	4	8	2	4	1	2
*Land Use & Design*								
Land use	44	88	32	64	3	6	9	18
Historic preservation	21	42	11	22	2	4	8	16
Farmland preservation	16	32	7	14	0	0	9	18
Design	13	26	5	10	1	2	7	14
Infill/Reuse	8	16	6	12	0	0	2	4
Mixed use	8	16	2	4	2	4	4	8
Smart growth	4	8	3	6	1	2	0	0
*Parks & Recreation*								
Parks/Recreation	45	90	23	46	5	10	17	34
Natural resources	37	74	19	38	5	10	13	26
Open space	30	60	16	32	5	10	9	18
Trails	10	20	3	6	3	6	4	8
*Other Relevant*								
Equity ^†^	31	62	15	30	6	12	10	20
Physical activity	2	4	0	0	1	2	1	2

*N* = 50. Addressed, item is addressed in state statute either as a mandate, conditional mandate, or encouraged; Mandated, item is required to be included in plan; Conditionally mandated, item is required to be included in plan if certain conditions are met; Encouraged, item is discussed in statute, but there is no language indicating it is required to be in the plan. All data are mutually exclusive. Item headings (italics) are for convenience only and do not reflect attributes of comprehensive plan state statutes themselves. See Appendix A for definitions of each element/topic category. ^†^ Equity is broadly defined here and includes language about housing and neighborhood quality.

**Table 2:** Prevalence and percent of states with laws addressing comprehensive plan primary elements, subsumed elements, and topics related to PA.

PA-Related Item	Primary Element		Subsumed Element		Topic	
	*n*	%	*n*	%	*n*	%
*Transportation*						
Transportation/Circulation	27	54	14	28	13	26
Streets	4	8	23	46	16	32
Public transportation	1	2	20	40	3	6
Bicycling	1	2	11	22	1	2
Pedestrian	0	0	8	16	2	4
Bicycle/Pedestrian	0	0	7	14	0	0
*Land Use & Design*						
Land use	36	72	9	18	11	22
Historic preservation	9	18	11	22	6	12
Design	4	8	5	10	4	8
Farmland preservation	3	6	9	18	5	10
Infill/Reuse	1	2	7	14	1	2
Smart growth	1	2	3	6	1	2
Mixed use	0	0	6	12	2	4
*Parks & Recreation*						
Natural resources	22	44	20	40	14	28
Parks/Recreation	11	22	29	58	20	40
Open space	7	14	18	36	12	24
Trails	0	0	8	16	2	4
*Other Relevant*						
Equity ^†^	2	4	2	4	11	22
Physical activity	0	0	2	4	0	0

*N* = 50. Primary element: Item referred to as element, plan, component, section, “objectives, policies, and programs”, or the item is among a list of same-hierarchy items that are referred to as elements, plans, components, sections, “objectives, policies, and programs”. Subsumed element: Included as part of what an element, plan, component, or section shall or may include. Topic: Items that may or must be included but are not primary or subsumed elements. Data are not mutually exclusive. Item headings (italics) are for convenience only and do not reflect attributes of comprehensive plan state statutes themselves. See Appendix A for definitions of each element/topic category. ^†^ Equity is broadly defined here and includes language about housing and neighborhood quality.

**Table 3:** Prevalence of states with population-conditional mandates for PA-related items, compared to prevalence of states addressing the item in state statute.

PA-Related Item	# of States with Population-Conditional Mandate on Item	# of States that Address Item ^1^	% of States that Address Item that Have a Population-Conditional Mandate for Item ^2^
*Transportation*			
Transportation/Circulation	5	40	12.5
Streets	5	37	13.5
Public transportation	3	23	13.0
Bicycling	2	11	18.2
Bicycle/Pedestrian	2	7	28.6
Pedestrian	0	9	0.0
*Land Use & Design*			
Land Use	6	44	13.6
Historic preservation	3	21	14.3
Mixed use	2	8	25.0
Design	2	13	15.4
Smart growth	1	4	25.0
Farmland preservation	0	16	0.0
Infill/Reuse	0	8	0.0
*Parks & Recreation*			
Parks/Recreation	7	45	15.6
Open space	6	30	20.0
Natural resources	5	37	13.5
Trails	2	10	20.0
*Other Relevant*			
Equity ^†^	5	31	16.1
Physical activity	0	2	0.0

^1^ Column “Overall—n” from Table 1. ^2^ # of states with population-conditional mandate on item/# of states that address item × 100. Item headings (italics) are for convenience only and do not reflect attributes of comprehensive plan state statutes themselves. See Appendix A for definitions of each element/topic category. ^†^ Equity is broadly defined here and includes language about housing and neighborhood quality.

**Table 4:** Prevalence and percent of states with statutes addressing comprehensive plan elements or topics related to PA, by state-level rurality.

	State-Level Rurality ^1^						
PA-Related Item	Least Rural (*n* = 16)		Mixed Rural/Urban (*n* = 17)		Most Rural (*n* = 17)		
	*n*	%	*n*	%	*n*	%	Fisher’s Exact ^2^ *p*-Value
*Transportation*							
Transportation/Circulation	16	100.0	12	70.6	12	70.6	**0.038**
Streets	12	75.0	13	76.5	12	70.6	1.000
Public transportation	11	68.8	7	41.2	5	29.4	**0.074**
Bicycling	5	31.3	4	23.5	2	11.8	0.401
Pedestrian	5	31.3	3	17.7	1	5.9	0.154
Bicycle/Pedestrian	4	25.0	1	5.9	2	11.8	0.269
*Land Use & Design*							
Land use	16	100.0	13	76.5	15	88.2	0.145
Historic preservation	8	50.0	6	35.3	7	41.2	0.721
Farmland preservation	7	43.8	5	29.4	4	23.5	0.478
Mixed use	7	43.8	1	5.9	0	0.0	**0.001**
Design	6	37.5	3	17.7	4	23.5	0.436
Infill/Reuse	6	37.5	1	5.9	1	5.9	**0.023**
Smart growth	4	25.0	0	0.0	0	0.0	**0.008**
*Parks & Recreation*							
Parks/Recreation	14	87.5	15	88.2	16	94.1	0.860
Natural resources	14	87.5	12	70.6	11	64.7	0.363
Open space	12	75.0	7	41.2	11	64.7	0.141
Trails	5	31.3	1	5.9	4	23.5	0.170
*Other Relevant*							
Equity ^†^	13	81.3	10	58.8	8	47.1	0.144
Physical activity	2	8.0	0	0.0	0	0.0	**0.098**

^1^ State-level rurality is defined as the percentage of the state population living outside of 2010 U.S. Census Bureau Urban Areas, categorized by tertile. ^2^ Fisher’s exact tests here test the null hypothesis that the percentage of states that include each item in their comprehensive planning statute do not differ between the three levels of rurality (i.e., that the gray columns do not differ). Fisher’s exact scores bolded for *p* < 0.10. Item headings (italics) are for convenience only and do not reflect attributes of comprehensive plan state statutes themselves. See Appendix A for definitions of each element/topic category. ^†^ Equity is broadly defined here and includes language about housing and neighborhood quality.

## Data Availability

The data presented in this study will be openly available in FigShare at DOI https://doi.org/10.6084/m9.figshare.15057468 after a 1-year embargo. Please contact the corresponding author for requests to access the data before the 1-year embargo has expired. The codebook for the dataset can be found at https://doi.org/10.6084/m9.figshare.15057426.

## Appendix A

**Table A1:** Coded language for each physical activity (PA)-related item identified in state statutes.

Item	Coded Language
*Transportation*	
Bicycle/Pedestrian	“bicycle and pedestrian ways” “bicycle, pedestrian facilities” “pedestrian and bicycle travel” “bicycling and pedestrian access and travelways” “paths for bicycles and pedestrians” “pedestrian and bicycle projects” “pedestrian and bikeway systems” “pedestrian and bicycle component”
Bicycling	Bicycle accommodations Bicycle facilities Bicycle paths Bicycle routes Bicycling routes Bikeways Multimodal transportation network that meets the needs of bicyclists
Pedestrian	Multimodal transportation network that meets the needs of pedestrians Pedestrian Pedestrian accommodations Pedestrian-oriented development Pedestrian ways Walking
Public transportation	Ground rapid transit systems Transit Mass Transit Mass Transportation Multimodal transportation network that meets the needs of users of public transportation Public Transit Public transportation facilities
Streets	Arterials Boulevards Freeways Highways Master street plan Parkways Public ways Rights-of-way Roads Road network Streets Thoroughfares
Transportation/Circulation	Circulation Circulation plan Mobility Multimodal circulation Multimodal transportation Plan for movement of people and goods Traffic circulation and transportation systems Transportation Transportation facilities Transportation plan Transportation routes Transportation system Transportation terminals and lines
*Land Use*	
Land use	Designation of areas for various types of public and private development and use Land and water use Land classification and utilization Land use Land uses Land utilization Use of land Uses of land
Design	Aesthetics Characteristics and community aesthetics important to future development Civic design Community design Design for subdivisions and unimproved land and areas subject to redevelopment Design guidelines Urban design Urban form and design
Infill/Reuse	Infill Redevelopment of vacant sites Reuse
Mixed use	Mix of uses Mixed land use Mixed use Mixture of land uses
Smart growth	Compact development Compact form development Discourage urban sprawl Smart growth
Farmland preservation	Agricultural preservation Agriculture protection area Conservation and restoration of existing farmlands Consideration of areas most suited for agricultural uses Discouragement of incompatible development in rural areas, including identifying critical rural areas Farmland preservation Preservation and protection of agricultural resources Preservation of character and density of rural neighborhoods Preservation of prime agricultural lands Preserves agricultural areas and activities, including prime farmlands and soils Protection of prime agricultural and forestlands Protection of agricultural land Protection of agricultural resources Protection of rural character of an area
Historic preservation	Areas; sites; and structures of historical, archeological, and architectural significance Conservation and restoration of historical resources Distribution and suitable uses of land, including historic areas Historic district boundaries/designated historically significant properties meriting protection Historic preservation Historical and archeological resources Historical preservation Identification of historically significant and other housing for purposes of conservation Preservation of historical features, sites, and monuments Preservation of rare and irreplaceable historic features and resources Preservation of historic places Protection of historic resources
*Parks & Recreation*	
Parks/Recreation	Parkland Parks Parks and recreation Playfields Playgrounds Recreation Recreational and tourism uses Recreational facilities Recreational land uses Recreational resources
Open space	Open development areas Open space areas Open spaces
Trails	Hiking trails Paths Riding trails Trail systems Trails Trailways
Natural resources	Areas; sites; and structures of ecological and wildlife significance Conservation Conservation and preservation of traprock and other ridgelines Conservation easements Conservation of forest lands Conservation of living and nonliving coastal zone resources Conservation of energy, water, soil, and agricultural and mineral resources Conservation of land and other irreplaceable natural resources Conservation of natural environment Conservation of natural resources Conservation of water and energy Conservation of water resources Conservation programs Conservation use and protection of natural resources Conserve significant natural resources Efficient use of energy Endangered or threatened species Energy conservation Minimizing development in sensitive shoreland areas Natural resources Nature preserves, wildlife management areas, and national forests Preservation Preservation of natural features, sites, and monuments Preservation of rare and irreplaceable natural areas Protection of critical waterfront areas Protection of environmental assets Protection of significant natural resource areas Protection of sensitive areas Protection of the quality and quantity of groundwater Renewable energy Sensitive areas Soil conservation Water conservation
*Other Relevant*	
Physical activity	Physical activity
Equity	Affordable housing Cost-burdened households Disabled Persons Disadvantaged community Disadvantaged populations Equal provision for the housing needs of all segments of the community regardless of race, color, creed, or economic level Elderly Elimination of substandard dwelling conditions Elimination of slums/blighted areas Housing affordability Housing quality, variety, and affordability Housing needs of residents earning less than 80% of the area median income Improvement of housing quality, variety, and affordability Improvement of housing standards Low-cost conventional housing Low Income Persons/Households Moderate income housing Older persons Persons with a disability Populations without automobiles/communities with limited [transportation] mode choice Redevelopment or rehabilitation of blighted areas Rehabilitation of housing in declining neighborhoods Replanning of blighted districts and slum areas Special housing needs (elderly, disabilities, large families, farmworkers, families with female heads of households, and families and persons in need of emergency shelter) Special needs Special needs of the transportation disadvantaged Special needs housing Special populations Unserved broadband areas

## Appendix B

**Table A2:** Population-based conditions on comprehensive planning mandates and rules addressing comprehensive plan elements or topics related to physical activity (PA), organized by state-level rurality.

State	Conditions for Planning Mandates	Conditions for Elements/Topics
*Least Rural*		
Arizona		Counties with 200,000+ population; otherwise encouraged. Counties with 125,000+ population; otherwise encouraged. Cities with 50,000+ population; otherwise encouraged. Cities and towns with 2500–10,000 population that have had a population increase average >2% per year for a 10-year period before the most recent US census AND cities and towns with >10,000 population; otherwise encouraged.
Colorado	(I) City & county or county with 10,000+ population and a population increase of either (a) 10%+ from 1994 to 1999, or (b) 10%+ during any 5-year period ending in 2000 or any subsequent year, AND (II) city & county or county with 100,000+ population; otherwise encouraged.Municipalities with 2000+ population that is wholly or partially subject to the requirements above.	(I) City and county or county with 10,000+ population and a population increase of either (a) 10%+ from 1994 to 1999, or (b) 10%+ during any 5-year period ending in 2000 or any subsequent year; AND (II) city and county or county with 100,000+ population; AND (III) the counties of Clear Creek, Gilpin, Morgan, and Pitkin. Municipalities with 2000+ population that is wholly or partially subject to the requirements above.
Florida		Municipalities with 50,000+ population. Counties with 75,000+ population. Local governments with >50,000 population.
Massachusetts	(I) Towns with 10,000+ population, and (II) towns with a planning board. Does not apply to Boston. Otherwise encouraged.	
Nevada	Counties with 45,000+ population AND counties of less than 45,000 population if they have a planning commission Cities with 25,000+ population AND cities with less than 25,000 population if they have a planning commission	Counties with 700,000+ population; otherwise encouraged. Counties with 100,000+ population; otherwise encouraged.
Utah		(I) Cities of the first (population 100,000+), second (population 65,000–99,999), third (population 30,000–64,999), or fourth (population 10,000–29,999) class; AND (II) cities of the fifth (population 1000–9999) class with 5000+ population, if the city is located within a county of the first (population 1,000,000+), second (population 175,000–999,999), or third (population 40,000–174,999) class; AND (III) metro townships with 5000+ population
Washington	(I) Counties that have a planning agency (i.e., a planning commission or a planning department) AND (II) counties (and the cities located therein) with 50,000+ population that have had population increase by >17% in the previous 10 years, AND (III) counties (and the cities located therein) that have had a population increase by >20% in the previous 10 years; otherwise encouraged. Cities located within counties that fulfill the above requirements AND cities in counties that opt-in to Growth Management Area planning.	(I) Counties (and the cities located therein) that opt-in to Growth Management Area planning, AND (II) counties (and the cities located therein) with 50,000+ population that have had a population increase by >17% in the previous 10 years, AND (III) counties (and the cities located therein) that have had a population increase by >20% in the previous 10 years.
*Mixed Rural/Urban*		
Delaware		Municipalities with 2000+ population.
Minnesota	Counties with <300,000 population and a planning commission; otherwise encouraged for counties <300,000.	
Nebraska	(I) Metropolitan and primary class cities (population 100,001+), AND (II) first and second class cities (population 801–100,000) that have adopted and not amended a zoning ordinance prior to 17 May 1967	Primary (population 100,001–299,000) class cities. Primary (population 100,001–299,000), first (population 5001–100,000), and second (population 801–5000) class cities. Metropolitan (population 300,000+), primary (population 100,001–299,000), first (population 5001–100,000), and second (population 801–5000) class cities.
Pennsylvania	Counties second through eighth class (counties < 1,500,000 population)	
*Most Rural*		
Kentucky	Counties with 300,000+ population, a consolidated local government, and a planning unit; otherwise encouraged.	
Oklahoma	Counties with 500,000+ population and a planning commission.	Cities with >200,000 population.

Where there are multiple bullet points for a state, different items had different population-based conditions and/or there were different conditions for counties and municipalities.
